# Eco-Friendly Synthesis and Antiproliferative Evaluation of Some Oxygen Substituted Diaryl Ketones

**DOI:** 10.3390/molecules18089818

**Published:** 2013-08-16

**Authors:** Paola Arenas, Andrés Peña, David Ríos, Julio Benites, Giulio G. Muccioli, Pedro Buc Calderon, Jaime A. Valderrama

**Affiliations:** 1 Facultad de Ciencias de la Salud, Universidad Arturo Prat, Casilla 121, Iquique 1100000, Chile; 2 Instituto de EtnoFarmacología (IDE), Universidad Arturo Prat, Casilla 121, Iquique 1100000, Chile; 3 Bioanalysis and Pharmacology of Bioactive Lipids Laboratory, Louvain Drug Research Institute, Université Catholique de Louvain, 72 Avenue E. Mounier, BPBL 7201, 1200 Brussels, Belgium; 4 Toxicology and Cancer Biology Research Group, Louvain Drug Research Institute, Université Catholique de Louvain, 73 Avenue E. Mounier, GTOX 7309, 1200 Brussels, Belgium; 5 Facultad de Química, Pontificia Universidad Católica de Chile, Casilla 306, Santiago 6094411, Chile

**Keywords:** photo-Friedel Crafts acylation, diaryl ketones, green chemistry, antiproliferative activity

## Abstract

A broad variety of oxygen-substituted diaryl ketones has been synthesized by solar energy-induced Friedel Crafts acylations of 1,4-benzo- and 1,4-naphthoquinones with benzaldehydes. The *in vitro* antiproliferative properties of the photoproducts were assessed on prostate (DU-145), bladder (T24) and breast (MCF7) human-derived tumor cell lines and compared to non-tumor mouse fibroblasts (Balb/3T3). Among the tested compounds, it was found that those containing a 3,4,5-trimethoxyphenyl A-ring, such as **12** and **22** are more active on DU-145, with EC_50_ values of 1.2 and 5.9 μM, respectively. By comparing their effects on the three cancer cell lines, the analogue **22** has the best mean selective index (2.4).

## 1. Introduction

Acylhydroquinones are valuable building blocks of natural [1,2,3,4] and synthetic compounds endowed with a variety of biological properties [5,6,7,8,9,10,11,12,13,14,15,16,17,18,19,20]. The classic procedures to construct the diaryl ketone framework of acylhydroquinones are based on the Friedel Crafts acylation and Fries rearrangement. Despite the efficiency of these synthetic methods, they have some disadvantages such as the lack of atom economy, the use of hazardous environmental Lewis acids, namely BF_3_, AlCl_3_, TiCl_4_, or ZnCl_2_ [21,22,23,24,25,26], and limitations regarding the use of precursors containing oxygen acid-labile functional groups. The photo-Friedel Crafts acylation of quinones with aliphatic and aromatic aldehydes to prepare acylhydroquinones is a green and efficient alternative method with respect to the classic aforementioned acylation methods. It is noteworthy that several examples of the preparation of acylhydroquinones by photoacylation of 1,4-quinone with aldehydes have been reported [27,28,29,30,31,32,33,34], however, the scope of this method to the synthesis of oxygen-substituted diaryl ketones had received relatively little attention. 

**Figure 1:**
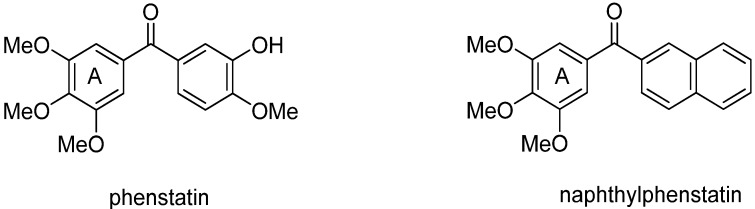
Structure of phenstatin and naphthylphenstatin.

Among the broad variety of synthetic diaryl ketones the oxygen-substituted members, named phenstatin [35] and naphthylphenstatin [36] (Figure 1), stand out by their biological activity as microtubule-targeting agents. Based on these precedents we wanted to examine the synthetic flexibility of the eco-friendly solar photoacylation of 1,4-benzo- and 1,4-naphthoquinone with substituted benzaldehydes to the synthesis of diverse oxygen-substituted diaryl ketones. Taking advantage of this potentially simple access to oxygen-substituted diaryl ketones we were also interested in evaluating the series for *in vitro* antiproliferative activity on cancer cells. The aim of this study is mainly directed towards broadening the use of simple and eco-friendly methodologies in the synthesis of new oxygen-substituted diaryl ketones as well as to contribute to the search for new biological active members of this series.

## 2. Results and Discussion

### 2.1. Chemistry

**Scheme 1:**
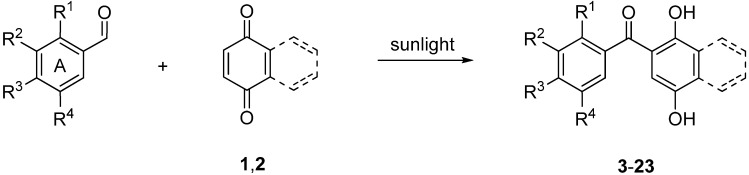
Photoacylation of quinones **1** and **2** with substituted benzaldehydes.

Based on our experience on the synthesis of heteroaroylhydroquinones by solar photoacylation of 1,4-benzo- and 1,4-naphthoquinone **1** and **2** with heteroarylcarbaldehydes in benzene [33], we initially examined the reaction of **1** and **2** with mono-substituted benzaldehydes. The reactions were carried out by using a 6.5 molar excess of the aldehyde with respect to the quinone. It is interesting to point out that molar excess of aldehyde is used to inhibit the dimerization of the quinone [30]. To avoid the use of hazardous benzene as the solvent, both the reaction of **1** and **2** with benzaldehyde and the liquid isomers of methyl- and methoxybenzaldehydes were accomplished using the appropriate aldehyde in excess as the solvent. In these experiments, performed by solar irradiation for 30 hours, the reaction mixtures were further submitted to column chromatography to give the respective photoproducts **3**–**8** and **14**–**18** (Scheme 1) in the 34%-77% yield range (Table 1). The formation yields of the products were determined on the basis of the initial and the amounts of the respective quinones recovered.

In parallel experiments, the above reactions were run in benzene in order to compare the yields of the photo-acylation reactions with and without this solvent. The results of these assays are collected in Table 1. The data indicate that the photoacylation reactions of quinones **1** and **2** give higher yields in benzene solvent than in excess aldehyde.

The photoacylation of quinones **1** and **2** with the solid di- and trisubstituted benzaldehydes were carried out in benzene under the above mentioned solar irradiation conditions. The treatment provides access to the corresponding photoproducts **9**–**13** and **19**–**23** in good yields (Table 1). The new diaryl ketones **7**, **9**–**13**, **15**, **19**–**21** and **23** were fully characterized by IR, ^1^H-, ^13^C-NMR and HRMS.

The synthesis of compounds **4** (65%), **6** (79%), **8** (77%), **14** (57%) and **16** (70%) have been previously reported by photoacylation of **1** and **2** with the respective aldehydes in the presence of catalytic amounts of benzophenone and using artificial light irradiation [29]. According to the data in Table 1 better yields on these compounds are achieved by using the solar chemical procedure (method B). Selected *indoor* photoacylation experiments performed in benzene by using irradiation with fluorescent lamps indicate that the photoproducts were generated in low yields.

**Table 1:** Oxygen-substituted diaryl ketones **3**–**23** prepared by solar photoacylation. 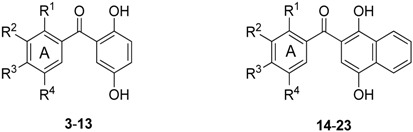

Photoproduct	Substituents				Yield (%) ^a^	
	R^1^	R^2^	R^3^	R^4^	Method A ^b^	Method B ^c^
**3**	H	H	H	H	77	91
**4 **	Me	H	H	H	52	80
**5**	H	Me	H	H	34	74
**6**	H	H	Me	H	58	82
**7**	H	OMe	H	H	38	74
**8**	H	H	OMe	H	59	79
**9**	OMe	H	H	OMe	-	70
**10**	H	H	OMe	OMe	-	70
**11**	H	OMe	OH	H	-	78
**12**	H	OMe	OMe	OMe	-	70
**13**	H	OMe	OH	OMe	-	70
**14**	Me	H	H	H	53	82
**15**	H	Me	H	H	41	69
**16**	H	H	Me	H	57	84
**17**	H	OMe	H	H	50	71
**18**	H	H	OMe	H	69	88
**19**	OMe	H	H	OMe	-	65
**20**	H	H	OMe	OMe	-	63
**21**	H	H	OH	OMe	-	73
**22**	H	OMe	OMe	OMe	-	60
**23**	H	OMe	OH	OMe	-	66

^a^ Isolated by column chromatography; ^b^ Method A: the reaction was carried out using **1** or **2** (1 equiv.) and the aldehyde (7.5 equiv.) without benzene; ^c^ Method B: the reaction was carried out using **1** or **2** (1 equiv.), the aldehyde (7.5 equiv.) and benzene as the solvent.

### 2.2. *In Vitro* Antiproliferative Activity of Diaryl Ketones ***3**–**23*** again Select Cancer Cell Lines

The oxygen-substituted diaryl ketones **3**–**23** were evaluated for their *in vitro* antiproliferative activity on a panel of four cell lines, including non-tumor fibroblasts (Balb/3T3) and three human-derived tumor cell lines, namely DU-145 (prostate), T24 (bladder) and MCF7 (breast), using the conventional microculture tetrazolium reduction assay [37].

**Table 2:** *In vitro* inhibitory effect of compounds **3**–**23** on the proliferation of the human-derived tumor cell lines: T24 (bladder), DU-145 (prostate) and MCF7 (breast) and the non-tumor fibroblasts (Balb/3T3). 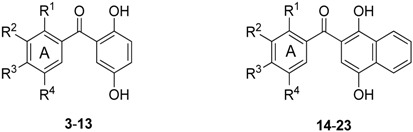

					EC_50_ ± SEM ^a^ (μM)				
N°	R^1^	R^2^	R^3^	R^4^	T24	DU-145	MCF-7	BALB/3T3	MSI ^b^
**3**	H	H	H	H	186.4 ± 20.4	205.0 ± 21.5	189.8 ± 19.2	38.5 ± 5.5	0.20
**4**	Me	H	H	H	172.5 ± 13.2	172.5 ± 19.7	180.7 ± 15.8	183.9 ± 17.6	1.06
**5**	H	Me	H	H	202.3 ± 15.9	186.2 ± 17.8	225.7 ± 24.2	100.3 ± 9.5	0.49
**6**	H	H	H	Me	208.9 ± 19.5	100.8 ± 9.5	142.1 ± 15.3	78.3 ± 6.4	0.57
**7**	H	OMe	H	H	209.7 ± 20.5	166.1 ± 15.8	154.4 ± 12.3	117.6 ± 19.5	0.68
**8**	H	H	OMe	H	168.1 ± 12.2	170.2 ± 15.5	190.7 ± 17.5	85.2 ± 9.7	0.49
**9**	OMe	H	H	OMe	149.7 ± 18.4	130.3 ± 12.5	164.5 ± 13.8	43.7 ± 7.6	0.29
**10**	H	H	OMe	OMe	147.4 ± 19.2	140.0 ± 13.6	182.9 ± 17.7	35.4 ± 4.7	0.23
**11**	H	OMe	OH	H	154.5 ± 14.5	167.5 ± 15.9	176.7 ± 19.4	78.0 ± 9.3	0.47
**12**	H	OMe	OMe	OMe	3.6 ± 1.4	1.2 ± 0.6	53.6 ± 6.7	1.5 ± 0.4	0.57
**13**	H	OMe	OH	OMe	>350	112.8 ± 10.7	>350	19.1 ± 3.1	-
**14**	Me	H	H	H	143.3 ± 9.5	143.8 ± 17.7	152.3 ± 14.5	225.5 ± 25.4	1.54
**15**	H	Me	H	H	136.8 ± 11.7	147.4 ± 15.9	144.9 ± 12.7	142.4 ± 13.9	0.99
**16**	H	H	Me	H	134.6 ± 12.8	158.3 ± 13.7	133.8 ± 14.9	138.9 ± 15.2	0.98
**17**	H	OMe	H	H	133.3 ± 12.4	139.4 ± 11.3	127.9 ± 13.6	109.1 ± 11.1	0.82
**18**	H	H	OMe	H	126.8 ± 10.6	77.5 ± 6.5	124.9 ± 10.2	107.0 ± 12.3	1.03
**19**	OMe	H	H	OMe	129.8 ± 10.1	128.7 ± 17.2	>310	100.3 ± 8.9	0.77
**20**	H	H	OMe	OMe	46.8 ± 5.1	61.8 ± 5.6	12.2 ± 3.8	2.8 ± 0.6	0.11
**21**	H	H	OH	OMe	86.6 ± 9.6	89.0 ± 7.3	15.7 ± 4.6	15.7 ± 3.9	0.45
**22**	H	OMe	OMe	OMe	13.5 ±1.9	5.9 ± 0.8	20.2 ± 4.5	25.1 ± 3.9	2.45
**23**	H	OMe	OH	OMe	112.0 ± 9.3	113.5 ± 9.5	130.2 ± 14.8	99.6 ± 7.5	0.84
DOX^c^	-	-	-	-	0.65 ± 0.07	0.42 ± 0.03	0.33 ± 0.05	0.19 ± 0.01	0.44
MIT^d^	-	-	-	-	42.2 ± 5.8	14.3 ± 2.6	16.8 ± 2.9	27.3 ± 3.3	1.39

^a^ Data represent EC_50_ mean values ± SEM of at least three different experiments; ^b^ MSI: Mean Selective Index = EC_50_ values fibroblasts/EC_50_ values tumor cells; ^c^ DOX: doxorubicin; ^d^ MIT: mitomycin C.

Table 2 summarizes the data from these evaluations: it shows the EC_50_ values (µM) of the respective benzo- and naphthohydroquinone derivatives. These values were calculated from their effects on MTT reduction in three cancer cell lines and Balb/3T3 non transformed mouse fibroblasts as a function of their concentration during 48 h of incubation. All three cancer cell lines were similarly sensitive to these compounds. With rather few exceptions, the members containing the dihydroxyphenyl fragment, such as **3**–**13**, were in general less active than their corresponding naphthyl analogues **14**–**23** when tested against cancer cells, but just the opposite was observed in non-transformed fibroblasts. The data in Table 2 showed that compounds **12** and **22** appear as the most potent members of the series with lower EC_50_ values against T24 and DU-145 with respect to the reference drug mitomycin C. The biological activity differences of compounds **12** and **22** with respect to their analogues could be attributed to the 3,4,5-trimethoxy substitution on the A-phenyl ring. According to literature precedents, the 3,4,5-trimethoxyphenyl ring is considered essential for the antitubulin activity of a broad variety of antimitotic compounds [36,38,39,40,41,42,43,44,45]. Nevertheless, it should be noted that the EC_50_ values of compounds **12** and **22** are two orders of magnitude lower than to that reported to phenstatin when tested in the NCI screen [35] showing a mean panel GI_50_ (growth inhibitory) value of 6.01 × 10^−8^ M. In addition to the tendency showing that dihydroxynaphthyl analogues are more active than the dihydroxyphenyl derivatives, the vast majority of compounds did not have an adequate selectivity (Table 2), that is they affect both cancer and non-tumor cells in a similar way. In this context, only compound **22** have a good mean selectivity index (2.45).

## 3. Experimental

### 3.1. General

All reagents were commercially available reagent grade and were used without further purification. Melting points were determined on a Stuart Scientific SMP3 apparatus and are uncorrected. The IR spectra were recorded on an FT Bruker spectrophotometer using KBr disks, and the wave numbers are given in cm^−1^. ^1^H-NMR spectra were run on Bruker AM-200 and AM-400 instruments in deuterochloroform (CDCl_3_) and dimethyl sulfoxide-d_6_ (DMSO-*d_6_*). Chemical shifts are expressed in ppm downfield relative to tetramethylsilane (TMS, δ scale), and the coupling constants (*J*) are reported in Hertz. ^13^C-NMR spectra were obtained in CDCl_3_ + DMSO-*d_6_* at 50 and 100 MHz. Chemical shifts are reported in δ ppm downfield from TMS, and *J*-values are given in Hertz. HRMS data were obtained on Thermo Finnigan mass spectrometer, model MAT 95XP and LTQ-Orbitrap mass spectrometer (Thermo-Fisher Scientific) with the analysis performed using an APCI source operated in positive mode. Silica gel Merck 60 (70–230 mesh) was used for preparative column chromatography and TLC aluminum foil 60F_254_ for analytical TLC. The solar irradiation experiments were performed at the Canchones Experimental Center in Iquique/Chile (latitude 20°26′43.80"S, 990 m above sea level), located in the Atacama Desert.

### 3.2. Chemistry

#### General Procedure for Photoacylation of **1** and **2** with Substituted Benzaldehydes in the Absence of Benzene (Method A)

Quinone **1** or **2** (1 mmol) and the liquid aldehyde (7.5 mmol), were placed into a test tube, nitrogen was bubbled through the solution for 2 min and then the tube was sealed with a septum. The mixture was irradiated for six days (total illumination time of 30 h), under solar radiation conditions in the range 800–1100 Watts/m^2^ (November–March). The mixture reaction was chromatographed on silica gel (3:1 petroleum ether/ethyl acetate) to give pure samples of the corresponding diaryl ketones. The remaining precursors were recovered to be used in further preparations.

#### General Procedure for Photoacylation of **1** and **2** with Substituted Benzaldehydes in Benzene (Method B).

A 100 mL benzene solution of the required quinone **1** or **2** (1 mmol) and the substituted benzaldehyde (7.5 mmol), was placed into the outer jacket of a Liebig condenser type. The solution was bubbled with nitrogen (2 min), the flask was sealed with a septum and then irradiated for six days (total illumination time of 30 h), under solar radiation conditions in the range 800–1100 Watts/m^2^ (November–March). The solvent was evaporated under reduced pressure and the residue was chromatographed on silica gel (3:1 petroleum ether/ethyl acetate). The starting aldehyde and the solvent were recovered and employed in the next batches.

*(2,5-Dihydroxyphenyl)(phenyl)methanone* (**3**). This compound was prepared from quinone **1** and benzaldehyde and was isolated in 77 and 91% yield by following methods A and B, respectively; orange solid, mp 121–122 °C (lit. [46]: 125–126 °C). IR (KBr) ν_máx_ cm^–1^: 3456 (O-H), 3358 (O-H), 1637 (C=O); ^1^H-NMR (CDCl_3_): δ 6.98 (m, 2H, 4'-H + 5-OH), 7.03 (s, 1H, 6-H), 7.11 (d, 2H, *J* = 7.2 Hz, 2'-H + 6'-H), 7.19 (m, 2H, 3'-H + 5'-H), 8.10 (d, 1H, *J* = 7.8 Hz, 3-H or 4-H), 8.21 (d, 1H, *J* = 7.8 Hz, 4-H or 3-H), 11.42 (bs 1H, 2-OH); ^13^C-NMR (CDCl_3_): δ 119.6, 119.9, 123.4, 125.5, 129.1, 129.5, 129.7, 130.4, 130.6, 132.7, 138.9, 149.9, 206.1; HRMS (M^+^): *m/z* calcd for C_13_H_10_O_3_: 214.06299; found: 214.06189.

*(2,5-Dihydroxyphenyl)(2'-methylphenyl)methanone* (**4**). This compound was prepared from quinone **1** and 2-methylbenzaldehyde in 52 and 80% yield following methods A and B, respectively; yellow solid, mp 104–105 °C (lit. [47]: 106–108 °C). IR (KBr) ν_máx_ cm^–1^: 3287 (O-H), 1638 (C=O); ^1^H-NMR (CDCl_3_): δ 2.29 (s, 3H, Me), 4.76 (s, 1H, 5-OH), 6.71 (d, 1H, *J* = 3.0 Hz, 6-H), 6.95 (d, 1H, *J* = 8.9 Hz, 3-H), 7.04 (dd, 1H, *J* = 8.9, 3.0 Hz, 4-H), 7.27 (m, 3H, 3'-H + 4'-H + 6'-H), 7.39 (m, 1H, 5’-H), 11.81 (s, 1H, 2-OH); ^13^C-NMR (CDCl_3_): δ 19.6, 118.1, 119.3, 119.5, 125.4, 125.5, 127.3, 130.2, 130.9, 135.4, 137.7, 147.4, 157.5, 203.9; HRMS (M^+^): *m/z* calcd for C_14_H_12_O_3_: 228.07864; found: 228.07767.

*(2,5-Dihydroxyphenyl)(3'-methylphenyl)methanone* (**5**). This compound was prepared from **1** and 3-methylbenzaldehyde in 34 and 74% yield following methods A and B, respectively; yellow solid, mp 119–120 °C (lit. [47]: 114–116 °C). IR (KBr) ν_máx_ cm^–1^: 3285 (O-H), 1637 (C=O); ^1^H-NMR (CDCl_3_): δ 2.36 (s, 3H, Me), 5.82 (s, 1H, 5-OH), 6.91 (m, 1H, 3-H or 4-H), 7.01 (m, 2H, 4-H or 3-H + 6-H), 7.31 (m, 2H, 4'-H + 5'-H or 5'-H + 6'-H), 7.38 (d, 1H, *J* = 7.2 Hz, 4'-H or 6'-H), 7.41 (s, 1H, 2'-H), 11.63 (s. 1H, 2-OH); ^13^C-NMR (CDCl_3_): δ 21.4, 118.6, 118.9, 119.1, 124.9, 126.2, 128.2, 129.4, 132.9, 137.6, 138.4, 147.4, 157.0, 201.7; HRMS (M^+^): *m/z* calcd for C_14_H_12_O_3_: 228.07864; found: 228.07809.

*(2,5-Dihydroxyphenyl)(4'-methylphenyl)methanone* (**6**). This compound was prepared from **1** and 4-methylbenzaldehyde in 58 and 82% yield following methods A and B, respectively; yellow solid, mp 135–136 °C. IR (KBr) ν_máx_ cm^−1^: 3442 (O-H), 1629 (C=O); ^1^H-NMR (CDCl_3_): δ 2.40 (s, 3H, Me), 5.44 (s, 1H, 5-OH), 6.93 (d, 1H, *J* = 7.6 Hz, 3-H), 7.02 (m, 2H, 4-H + 6H), 7.24 (d, 2H, *J* = 8.1 Hz, 2'-H + 3'-H or 5'-H + 6'-H), 7.54 (d, 2H, *J* = 8.1 Hz, 3'-H + 2'-H or 6'H + 5'-H), 11.58 (s, 1H, 2-OH); ^13^C-NMR (CDCl_3_): δ 21.6, 118.4, 119.0, 119.2, 124.7, 129.1 (2 × C), 129.4 (2 × C), 134.9, 142.9, 147.3, 157.0, 201.7; HRMS (M^+^): *m/z* calcd for C_14_H_12_O_3_: 228.07864; found: 228.07831.

*(2,5-Dihydroxyphenyl)(3'-methoxyphenyl)methanone* (**7**). This compound was prepared from **1** and 3-methoxybenzaldehyde in 38 and 54% yield following methods A and B, respectively; yellow solid, mp 97–98 °C. IR (KBr) ν_máx_ cm^–1^: 3344 (O-H), 1635 (C=O); ^1^H-NMR (CDCl_3_): δ 3.80 (s, 3H, OMe), 5.80 (s, 1H, 5-OH), 6.91 (d, 1H, *J* = 9.8 Hz, 3-H or 4-H) 7.02 (m, 2H, 4-H or 3-H + 6-H), 7.06 (dd, 1H, *J* = 8.2, 2.2, Hz 4'-H or 6'-H), 7.14 (d, 1H, *J* = 2.2 Hz 2'-H), 7.16 (d, 1H, *J* = 7.9 Hz, 6'-H or 4'-H), 7.32 (t, 1H, *J* = 7.9 Hz, 5'-H), 11.56 (s, 1H-2-OH); ^13^C-NMR (CDCl_3_): δ 55.5, 113.9, 118.1, 118.4, 118.8, 119.2, 121.6, 125.1, 129.4, 138.8, 147.5, 157.0, 159.4, 201.2; HRMS (M^+^): *m/z* calcd for C_14_H_12_O_4_: 244.07356; found: 244.07361.

*(2,5-Dihydroxyphenyl)(4'-methoxyphenyl)methanone* (**8**). This compound was prepared from **1** and 4-methoxybenzaldehyde, in 58 and 79% yield according methods A and B, respectively; yellow solid, mp 144–145 °C. IR (KBr) ν_máx_ cm^−1^: 3343 (O-H), 1631 (C=O); ^1^H-NMR (CDCl_3_): δ 3.87 (s, 3H, OMe), 6.90 (d, 1H, *J* = 8.8 Hz, 3-H or 4-H), 6.97 (d, 2H, *J* = 8.8 Hz, 2'-H + 3'-H or 5'-H + 6'-H), 7.08 (m, 2H, 4-H or 3-H + 6-H), 7.72 (d, 2H, *J* = 8.8 Hz, 6'-H + 5'-H or 3'-H + 2'-H), 8.32(s, 1H, 5-OH), 11.38 (s, 1H, 2-OH); ^13^C-NMR (CDCl_3_): δ 55.5, 113.6, 115.9, 118.1, 118.7, 119.2, 122.4, 124.4, 130.4, 131.8, 148.7, 155.9, 162.8, 199.7; HRMS (M^+^): *m/z* calcd for C_14_H_12_O_4_: 244.07356; found: 244.07360.

*(2,5-Dihydroxyphenyl)(2',5'-dimethoxyphenyl)methanone* (**9**). This compound was prepared from **1** and 2,5-dimethoxybenzaldehyde in 70% yield (method B); yellow solid, mp 135–136 °C. IR (KBr) ν_máx_ cm^−1^: 3299 (O-H), 1637 (C=O); ^1^H-NMR (CDCl_3_ + DMSO-*d_6_*): δ 3.74 (s, 3H, OMe), 3.78 (s, 3H, OMe), 6.82 (s, 2H, 6-H + 6'-H), 6.87 (d, 1H, *J* = 8.8 Hz, 3-H), 6.96 (m, 2H, 3'-H + 4'-H), 7.06 (d, 1H, *J* = 8.8 Hz, 4-H), 8.39 (s, 1H, 5-OH), 11.60 (s, 1H, 2-OH); ^13^C-NMR (CDCl_3_ + DMSO-*d_6_*): δ 55.9, 56.4, 113.1, 113.8, 116.0, 116.8, 118.2, 118.4, 119.8, 125.6, 149.1, 150.6, 153.4, 156.3, 201.3; HRMS (M^+^): m/z calcd for C_15_H_14_O_5_: 274.08412; found: 274.08316.

*(2,5-Dihydroxyphenyl)(3,4-dimethoxyphenyl)methanone* (**10**). This compound was prepared from **1** and 3,4-dimethoxybenzaldehyde in 70% yield (method B); brown solid, mp 79–81 °C. IR (KBr) ν_máx_ cm^–1^: 3354 (O-H), 1630 (C=O); ^1^H-NMR (CDCl_3_ + DMSO-*d_6_*): δ 3.86 (s, 6H, 2 × OMe), 6.83 (s, 1H, 6'-H), 6.82 (s, 1H, 5'-H), 6.89 (d, 1H, *J* = 9.2 Hz, 3-H), 6.94 (s, 1H, 2'-H), 6.97 (d, 1H, *J* = 8.8 Hz, 4-H), 7.18 (s, 1H, 6-H), 8.69 (s, 1H, 5-OH), 11.34 (s, 1H, 2-OH); ^13^C-NMR (CDCl_3_ + DMSO-*d_6_*): δ 55.9, 60.5, 104.5, 111.2, 117.9, 118.4, 118.8, 122.5, 124.6, 133.0, 149.7, 149.8, 152.8, 155.7, 200.0; HRMS (APCI): [M+H]^+^ calcd for C_15_H_14_O_5_: 275.08412; found: 275.09072.

*(2,5-Dihydroxyphenyl)(4'-hydroxy-3'-methoxyphenyl)methanone* (**11**). This compound was prepared in 78% yield (method B) from **1** and 4-hydroxy-3-methoxybenzaldehyde; yellow solid, mp 221–222 °C. IR (KBr) ν_máx_ cm^–1^: 3329 (O-H), 1639 (C=O); ^1^H-NMR (CDCl_3_ + DMSO-*d_6_*): δ 3.77 (s, 3H, OMe), 6.86 (d, 1H, *J* = 8.0 Hz, 4-H or 3-H), 6.95 (d, 1H, *J* = 8.0 Hz, 3-H or 4-H), 7.03 (d, 1H, *J* = 8.8 Hz, 6'-H), 7.11 (s, 1H, 2'-H), 7.26 (d, 1H, *J* = 8.4 Hz, 5'-H), 7.31 (s, 1H, 6-H), 7.63 (s, 1H, 4'-OH), 8.89 (s, 1H, 5-OH), 11.10 (s, 1H, 2-OH); ^13^C-NMR (CDCl_3_ + DMSO-*d_6_*): δ 55.5, 107.1, 111.5, 112.2, 114.1, 121.7, 123.7, 125.4, 128.5, 143.7, 146.7, 149.7, 156.1, 198.9; HRMS (M^+^): *m/z* calcd for C_14_H_12_O_5_: 260.06847; found: 260.06764.

*(2,5-Dihydroxyphenyl)(3',4',5'-trimethoxyphenyl)methanone* (**12**). This compound was prepared in 70% yield (method B) from **1** and 3,4,5-trimethoxybenzaldehyde; yellow solid, mp 68–70 °C. IR (KBr) ν_máx_ cm^–1^:3445 (OH), 3200 (O-H), 1639 (C=O); ^1^H-NMR (CDCl_3_ + DMSO-*d_6_*): δ 3.81 (s, 9H, 3 × OMe), 6.81 (d, 1H, *J* = 8.8 Hz, 3-H), 6.87 (s, 2H, 2'-H + 6'-H), 7.0 (d, 1H, *J* = 8.8 Hz, 4-H), 7.07 (s, 1H, 6-H), 8.58 (s, 1H, 5-OH), 11.23 (s, 1H, 2-OH); ^13^C-NMR (CDCl_3_ + DMSO-*d_6_*): δ 56.3 (2 × C), 60.8, 107.0, 117.9, 118.7, 118.8, 124.9, 133.0, 141.3, 149.0 (2 × C), 149.9, 152.8, 156.0, 199.9; HRMS (M^+^): *m/z* calcd for C_16_H_16_O_6_: 304.09469; found: 304.09378.

*(2,5-Dihydroxyphenyl)(4'-hydroxy-3',5'-dimethoxyphenyl)methanone* (**13**). This compound was prepared in 70% (method B) from **1** and 4-hydroxy-3,5-dimethoxybenzaldehyde; yellow solid, mp 200–201 °C. IR (KBr) ν_máx_ cm^–1^: 3331(O-H), 1635 (C=O); ^1^H-NMR (CDCl_3_ + DMSO-*d_6_*): δ 3.92 (s, 6H, 2 × OMe), 6.87 (d, 1H, *J* = 9.0 Hz, 3-H), 7.03 (s, 3H, 6-H + 2'-H + 6'-H), 7,16 (d, 1H, *J* = 9.0 Hz, 4-H), 8.47 (s, 1H, 4-OH), 8.78 (s, 1H, 5-OH), 11.15 (s, 1H, 2-OH); ^13^C-NMR (CDCl_3_ + DMSO-*d_6_*): δ 56.0 (2 × C), 107.2 (2 × C), 117.3, 118.0, 118.9, 123.5, 127.5, 139.6, 146.8 (2 × C), 148.6, 154.7, 198.6; HRMS (M^+^): *m/z* calcd for C_15_H_14_O_6_: 290.07904; found: 290.07830.

*(1,4-Dihydroxynaphthalen-2-yl)(2'-methylphenyl)methanone* (**14**). This compound was prepared from quinone **2** and 2-methylbenzaldehyde in 53 and 82% yield following methods A and B, respectively; orange solid, mp 132–133 °C. IR (KBr) ν_máx_ cm^−1^: 3357 (O-H), 1638 (C=O); ^1^H-NMR (CDCl_3_): δ 2.23 (s, 3H, Me), 5.53 (s, 1H, 4-OH), 6.46 (s, 1H, 3-H), 7.17 (m, 3H, 3'-H + 4'-H + 6'-H), 7.26 (m, 1H, 5'-H), 7.58 (t, 1H, *J* = 7.6 Hz, 6-H or 7-H), 7.66 (t, 1H, *J* =7.6 Hz, 7-H or 6-H), 8.07 (d, 1H, *J* = 8.3 Hz, 5-H or 8-H), 8.50 (d, 1H, *J* = 8.3 Hz, 8-H or 5-H), 13.63 (s, 1H, 1-OH); ^13^C-NMR (CDCl_3_): δ 19.7, 107.5, 112.4, 121.9, 124.0, 125.4, 125.9, 126.7, 127.0, 129.8, 129.9, 130.3, 130.8, 135.2, 138.0, 143.1, 158.8, 203.7; HRMS (APCI): [M+H]^+^ calcd for C_18_H_14_O_3_: 279.09429; found: 279.10136.

*(1,4-Dihydroxynaphthalen-2-yl)(3'-methylphenyl)methanone* (**15**). This compound was prepared from quinone **2** and 3-methylbenzaldehyde in 41 and 69% yield according methods A and B, respectively; orange solid, mp 154–155 °C. IR (KBr) ν_máx_ cm^–1^: 3313 (O-H), 1633 (C=O); ^1^H-NMR (CDCl_3_): δ 2.44 (s, 3H, Me), 6.98 (s, 1H, 3-H), 7.39 (m, 2H, 4'-H or 6'-H + 2'-H), 7.51 (m, 2H, 6'-H or 4'-H + 5'-H), 7.56 (m, 1H, 6-H or 7-H), 7.66 (t, 1H, *J* = 7.6 Hz, 7-H or 6-H), 8.20 (d, 1H, *J* = 8.3 Hz, 5-H or 8-H), 8.46 (d, 1H, *J* = 8.3 Hz, 8-H or 5-H), 8.87 (s, 1H, 4-OH), 13.50 (s, 1H, 1-OH); ^13^C-NMR (CDCl_3_): δ 21.4, 107.4, 111.9, 122.3, 124.2, 125.8 (2 × C), 126.1, 127.9, 129.3, 129.5, 130.1, 132.0, 138.1, 138.5, 144.3, 157.4, 201.2; HRMS (APCI): [M+H]^+^ calcd for C_18_H_14_O_3_: 279.09429; found: 279.10124.

*(1,4-Dihydroxynaphthalen-2-yl)(4'-methylphenyl)methanone* (**16**). This compound was prepared from **2** and 4-methylbenzaldehyde in 57 and 84% yield according methods A and B, respectively; orange solid, mp 150–151 °C. IR (KBr) ν_máx_ cm^−1^: 3422 (O-H), 1635 (C=O); ^1^H-NMR (CDCl_3_): δ 2.43 (s, 3H, Me), 7.0 (s, 1H, 3-H), 7.30 (d, 2H, *J* = 7.8 Hz, 2'-H + 3'-H or 5'-H + 6'-H), 7.56 (t, 1H, *J* = 7.5 Hz, 6-H or 7-H), 7.64 (d, 2H, *J* = 7.8 Hz, 3'-H + 2'-H or 6'-H + 5'-H), 7.65 (m, 1H, 7-H or 6-H), 8.20 (d, 1H, *J* = 8.3 Hz, 5-H or 8-H), 8.47 (d, 1H, *J* = 8.3 Hz, 8-H or 5-H), 8.71 (s, 1H, 4-OH), 13.48 (s, 1H, 1-OH); ^13^C-NMR (CDCl_3_): δ 21.6, 107.6, 111.9, 122.3, 124.2, 125.9, 126.1, 128.9 (2 × C), 129.3 (2 × C), 129.4, 129.9, 135.8, 141.9, 144.2, 157.3, 200.8; HRMS (APCI): [M+H]^+^ calcd for C_18_H_14_O_3_: 279.09429; found: 279.10122.

*(1,4-Dihydroxynaphthalen-2-yl)(3'-methoxyphenyl)methanone* (**17**). This compound was prepared from **2** and 3-methoxybenzaldehyde in 50 and 71% yield according methods A and B, respectively; orange solid, mp 149–150 °C (lit. [34]: 142-144 °C). IR (KBr) ν_máx_ cm^–1^: 3344 (O-H), 1633 (C=O); ^1^H-NMR (CDCl_3_): δ 3.85 (s, 3H, OMe), 6.99 (s, 1H, 3-H), 7.08 (dd, 1H, *J* = 8.1, 2.0 Hz, 4'-H or 6'-H), 7.23 (s, 1H, 2'-H), 7.28 (m, 1H, 6'-H or 4'-H), 7.39 (t, 1H, *J* = 7.9 Hz, 5'-H), 7.56 (t, 1H, *J* = 7.6 Hz, 6-H or 7-H), 7.66 (t, 1H, *J* = 7.6 Hz 7-H or 6-H), 8.20 (d, 1H, *J* = 8.3 Hz, 5-H or 8-H), 8.47 (d, 1H, *J* = 8.3 Hz 8-H or 5-H), 8.65 (s, 1H, 4-OH), 13.46 (bs, 1H, 1-OH); ^13^C-NMR (CDCl_3_): δ 55.5, 107.3, 111.9, 113.7, 117.6, 121.4, 122.3, 124.3, 125.9, 126.2, 129.3, 129.6, 130.1, 139.8, 144.3, 157.7, 159.3, 200.7; HRMS (M^+^): *m/z* calcd for C_18_H_14_O_4_: 294.08921; found: 294.08854.

*(1,4-Dihydroxynaphthalen-2-yl)(4'-methoxyphenyl)methanone* (**18**). This compound was prepared from **2** and 4-methoxybenzaldehyde in 69 and 88% yield according methods A and B, respectively; yellow solid, mp 150-151°C (lit. [34]: 130–132 °C). IR (KBr) ν_máx_ cm^–1^: 3470 (O-H), 1631 (C=O); ^1^H-NMR (CDCl_3_): δ 3.86 (s, 3H, OMe), 6.97 (d, 2H, *J* = 8.7 Hz, 2'-H + 3'-H or 5'-H + 6'-H), 7.02 (s, 1H, 3-H), 7.56 (t, 1H, *J* = 7.4 Hz, 6-H or 7-H), 7.65 (t, 1H, *J* = 7.4 Hz, 7-H or 6-H), 7.75 (d, 2H, *J* = 8.7 Hz, 6'-H + 5'-H or 3'-H + 2'-H), 8.20 (d, 1H, *J* = 8.3 Hz, 5-H or 8-H), 8.47 (m, 2H, 8-H or 5-H + 4-OH), 13.43 (s, 1H, 1-OH); ^13^C-NMR (CDCl_3_): δ 55.5, 107.8, 111.9, 113.5, 122.3, 124.2, 125.9, 126.1, 129.3 (2 × C), 129.8, 130.9, 131.6 (2 × C), 144.1, 157.2, 162.4, 199.6; HRMS (APCI): [M+H]^+^ calcd for C_18_H_14_O_4_: 295.08921; found: 295.08059.

*(1,4-Dihydroxynaphthalen-2-yl)(2',5'-dimethoxyphenyl)methanone* (**19**). This compound was prepared from **2** and 2,5-dimethoxybenzaldehyde in 65% yield (method B); yellow solid, mp 160–161 °C. IR (KBr) ν_máx_ cm^–1^: 3389 (O-H), 1630 (C=O); ^1^H-NMR (CDCl_3_ + DMSO-*d_6_*): δ 3.75 (s, 3H, OMe), 3.79 (s, 3H, OMe), 6.70 (s, 1H, 6'-H), 6.90 (s, 1H, 3-H), 6.97 (m, 2H, 3'-H + 4'-H), 7.56 (t, 1H, *J* = 8.8 Hz, 6-H or 7-H), 7.66 (t, 1H, *J* = 8.8 Hz, 7-H or 6-H), 8.18 (d, 1H, *J* = 8.4 Hz, 8-H), 8.47 (s, 1H, 4-OH), 8.48 (d, 1H, *J* = 8.4 Hz, 5-H), 13.48 (s, 1H, 1-OH); ^13^C-NMR (CDCl_3_ + DMSO-*d_6_*): δ 56.0, 56.6, 107.4, 113.1, 113.2, 113.9, 117.0, 122.4, 124.5, 125.9, 126.2, 129.1, 129.7, 130.5, 144.4, 150.7, 153.5, 157.4, 200.8; HRMS (M^+^): *m/z* calcd for C_19_H_16_O_5_: 324.09978; found: 324.09914.

*(1,4-Dihydroxynaphthalen-2-yl)(3',4'-dimethoxyphenyl)methanone* (**20**). This compound was prepared from **2** and 3,4-dimethoxybenzaldehyde in 63% yield (method B); orange solid, mp 212–213 °C. IR (KBr) ν_máx_ cm^–1^: 3462 (O-H), 1638 (C=O); ^1^H-NMR (CDCl_3_ + DMSO-*d_6_*): δ 3.65 (s, 6H, 2 × OMe), 6.82 (s, 1H, 5'-H), 6.88 (s, 1H, 6'-H or 2'-H), 7.00 (s, 1H, 2'-H or 6'-H), 7.12 (s, 1H, 3-H), 7.56 (d, 1H, *J* = 6.8 Hz, 8-H), 8.19 (d, 1H, *J* = 6.8 Hz, 5-H), 8.46 (t, 2H, *J* = 8.4 Hz, 6-H + 7-H), 9.05 (s, 1H, 4-OH), 13.42 (s, 1H, 1-OH); ^13^C-NMR (CDCl_3_ + DMSO-*d_6_*): δ 56.0, 60.7, 104.7, 107.3, 111.0, 111.8, 122.3, 124.1, 126.0, 126.7, 128.7, 129.3, 129.9, 130.9, 144.3, 149.8, 152.9, 157.1, 199.9; HRMS (APCI): [M+H]^+^ calcd for C_19_H_16_O_5_: 325.09977; found: 325.09146.

*(1,4-Dihydroxynaphthalen-2-yl)(4'-hydroxy-3'-methoxyphenyl)methanone* (**21**). This compound was prepared from **2** and 4-hydroxy-3-methoxybenzaldehyde in 73% yield (method B); orange solid, mp 221–222 °C. IR (KBr) ν_máx_ cm^–1^: 3469 (O-H), 1635 (C=O); ^1^H-NMR (CDCl_3_ + DMSO-*d_6_*): δ 3.96 (s, 3H, OMe), 7.00 (d, 1H, *J* = 7.8 Hz, 8-H), 7.13 (s, 1H, 2'-H), 7.33 (d, 1H, *J* = 7.6 Hz, 5-H), 7.43 (s, 1H, 3-H), 7.56 (t, 1H, *J* = 7.8 Hz, 6-H or 7-H), 7.65 (t, 1H, *J* = 7.8 Hz, 7-H or 6-H), 8.20 (d, 1H, *J* = 8.0 Hz, 6'-H or 5'-H), 8.44 (d, 1H, *J* = 8.0 Hz, 5'-H o 6'-H), 8.81 (s, 1H, 4'-OH), 9.04 (s, 1H, 4-OH), 13.38 (s, 1H, 1-OH); ^13^C-NMR (CDCl_3_ + DMSO-*d_6_*): δ 55.9, 107.5, 112.0, 112.6, 114.5, 122.1, 123.9, 124.1, 125.7, 125.8, 128.9, 129.5, 129.7, 144.1, 147.1, 150.1, 156.5, 199.3; HRMS (M^+^): *m/z* calcd for C_18_H_14_O_5_: 310.08413; found: 310.08401.

*(1,4-Dihydroxynaphthalen-2-yl)(3',4',5'-trimethoxyphenyl)methanone* (**22**). This compound was prepared from **2** and 3,4,5-trimethoxybenzaldehyde in 60% yield (method B); brown solid, mp 189–191 °C. Mp and the spectral properties of **22** were in agree to those reported in literature [19].

*(1,4-Dihydroxynaphthalen-2-yl)(4'-hydroxy-3',5'-dimethoxyphenyl)methanone* (**23**). This compound was prepared from **2** and 4-hydroxy-3,5-dimethoxybenzaldehyde in 66% yield (method B); yellow solid, mp 173–174 °C. IR (KBr) ν_máx_ cm^–1^: 3346 (O-H), 1630 (C=O); ^1^H-NMR (CDCl_3_ + DMSO-*d_6_*): δ 3.95 (s, 6H, 2 × OMe), 7.08 (s, 2H, 2'-H + 6'-H), 7.16 (s, 1H, 3-H), 7.56 (t, 1H, *J* = 7.2 Hz, 6-H or 7-H), 7.66 (t, 1H, *J* = 7.2 Hz, 7-H or 6-H), 8.20 (d, 1H, *J* = 8.4 Hz, 8-H), 8.45 (d, 1H, *J* = 8.4 Hz, 5-H), 9.11 (s, 2H, 4-OH), 13.34 (s, 1H, 1-OH); ^13^C-NMR (CDCl_3_ + DMSO-*d_6_*): δ 56.3 (2 × C), 107.2 (2 × C), 111.8, 122.1, 122.5, 123.9, 125.7, 125.9, 128.6, 129.0, 129.5, 139.0, 144.2, 146.9, (2 × C), 149.3, 199.2; HRMS (M^+^): *m/z* calcd for C_19_H_16_O_6_: 340.09469; found: 340.09380.

### 3.3. Antiproliferative Assay

#### 3.3.1. Cell Lines and Culture Conditions

Human cancer cell lines (T24, DU-145, MCF7) were cultured in high-glucose Dulbecco's modified Eagle medium (Gibco, Grand Island, NY, USA) supplemented with 10% foetal calf serum, penicillin (100 U/mL), and streptomycin (100 μg/mL). Balb/3T3 cells (normal mouse fibroblasts) were cultured in the same medium, except that the foetal calf serum was replaced by 10% newborn calf serum. All cultures were kept at 37 °C in 95% air/5% CO_2_ at 100% humidity. Phosphate-buffered saline (PBS) was purchased from Gibco. Cells were incubated at the indicated times at 37 °C with or without hydroquinones at various concentrations.

#### 3.3.2. Cellular Assays

The cytotoxicity of the bis aryl ketones was assessed by following the reduction of MTT (3-(4,5-dimethylthiazolyl-2)-2,5-diphenyltetrazolium bromide) to formazan blue [37]. Briefly, cells were seeded into 96-well plates at a density of 10,000 cells/well for 24 h and then incubated for 48 h with or without the compounds. Cells were then washed twice with warm PBS and incubated with MTT (0.5 mg/mL) for 2 h at 37 °C. Incubation medium was thereafter discarded and the blue formazan crystals were solubilized by adding 100 μL DMSO/well. The colour solution was subsequently read at 550 nm. Results are expressed as % of MTT reduction compared to untreated control conditions. The calculation of EC_50_ values was performed by using GraphPad Prism software (San Diego, CA, USA).

## 4. Conclusions

We have extended the photo-Friedel Crafts acylation of 1,4-benzo- and 1,4-naphthoquinone with aldehydes to the synthesis of a significant number of oxygen-substituted diaryl ketones. The main advantages of this general procedure respect to other methods to construct oxygen-substituted diaryl ketone framework are the atom economy, simplicity, cheap and the chemical stability of the oxygen substituent of precursors and/or products. From the antiproliferative screening of the oxygen-substituted diaryl ketones, compounds **12** and **22** stand out by their biological activity levels on prostate DU-145 (EC_50_: 1.2 and 5.9 μM) and bladder T24 (EC_50_: 3.6 and 13.5 μM) cell lines, compared to those of the anticancer drug mitomycin C (EC_50_: 14.3 and 42.2 μM). Even though compound **22** displayed less potency than the analog **12**, it exhibited a better mean selective index. Although compounds **12** and **22** have EC_50_ values lower than to GI_50_ values reported for phenstatin, due to their structural similarity it may be hypothesized that inhibition of microtubule assembly is involved in the antiproliferative mechanism of these two compounds. Chemical modifications of compounds **12** and **22** directed to access to the scaffold of future active tubulin polymerization inhibitors are in progress.

## Supplementary Materials

Supplementary materials can be accessed at: http://www.mdpi.com/1420-3049/18/8/9818/s1.
