# Supplementary material for: Eco-Friendly Synthesis and Antiproliferative Evaluation of Some Oxygen Substituted Diaryl Ketones
Source: Molecules. 2013 Aug 16;18(8):9818–32. doi: 10.3390/molecules18089818 (PMC6270201; doi:10.3390/molecules18089818)
Supplement: Supplementary file 1 [file molecules-18-09818-s001.pdf]

Supplementary Materials

CHECK LIST

COMPOUNDS NUMBER	IR	^{1}H NMR	^{13}C NMR	HRMS	Mp
**3**	+	+	+	+	+
**4**	+	+	+	+	+
**5**	+	+	+	+	+
**6**	+	+	+	+	+
**7**	+	+	+	+	+
**8**	+	+	+	+	+
**9**	+	+	+	+	+
**10**	+	+	+	+	+
**11**	+	+	+	+	+
**12**	+	+	+	+	+
**13**	+	+	+	+	+
**14**	+	+	+	+	+
**15**	+	+	+	+	+
**16**	+	+	+	+	+
**17**	+	+	+	+	+
**18**	+	+	+	+	+
**19**	+	+	+	+	+
**20**	+	+	+	+	+
**21**	+	+	+	+	+
**22**	+	+	+	+	+
**23**	+	+	+	+	+
